# Characteristics, Attitudes and Preferences of an End-Stage Kidney Disease Population at the Beginning of an Exercise Program: A Pragmatic Multicenter Trial

**DOI:** 10.3390/jcm15041547

**Published:** 2026-02-15

**Authors:** Giovanni Piva, Francisco Labrador, Claudia Momenté, Nicola Lamberti, Anna Crepaldi, Alessio Di Maria, Yuri Battaglia, Alejandro Martin-Malo, Fabio Manfredini, Pablo Jesus Lopez-Soto, Alda Storari

**Affiliations:** 1 Department of Neuroscience and Rehabilitation, University of Ferrara, 44121 Ferrara, Italy; giovanni.piva@unife.it; 2 Instituto Maimónides de Investigación Biomédica de Córdoba (IMIBIC), 14004 Córdoba, Spainpablo.lopez@imibic.org (P.J.L.-S.); 3 Department of Nursing, Hospital Universitario Reina Sofía, 14004 Córdoba, Spain; 4 Department of Nursing, Pharmacology and Physiotherapy, Universidad de Córdoba, 14004 Córdoba, Spain; a.storari@ospfe.it; 5 Department of Medicine, University of Verona, 37134 Verona, Italy; claudia.momente@gmail.com (C.M.); yuri.battaglia@univr.it (Y.B.); 6 Unit of Nephrology, University Hospital of Ferrara, 44124 Ferrara, Italyalessio.dimaria@ospfe.it (A.D.M.); 7 Nephrology and Dialysis Unit, Pederzoli Hospital, 37019 Peschiera del Garda, Italy; 8 Instituto Maimónides de Investigación Biomédica de Córdoba (IMIBIC), Unidad de Gestión Clínica Nefrología, Reina Sofia University Hospital, Department of Medicine, University of Cordoba, 14004 Cordoba, Spain; alejandro.martin.sspa@juntadeandalucia.es; 9 Program of Vascular Rehabilitation and Exercise Medicine, University Hospital of Ferrara, 44124 Ferrara, Italy

**Keywords:** exercise, end-stage kidney disease, barriers, facilitator, attitudes, mobility

## Abstract

**Background/Objectives**: This multicenter nonrandomized pragmatic trial (NCT04282616), offering different options for active support by an exercise facilitator (EF) in the dialysis unit, aims to explore the attitudes and preferences of end-stage kidney disease (ESKD) patients in relation to their characteristics, capabilities and barriers. **Methods**: In six European dialysis units, an EF was set to offer to each ESKD patient able to walk four simple low-cost three-month interventions: (i) advised physical activity increase (U-PA-I); (ii) structured home-based walking exercise (S-HB-LI); (iii) in-hospital (pre/postdialysis) supervised walking and resistance low-intensity training (S-SU-LI); and (iv) performance assessment only (PPA). After collecting attitudes and perceptions of patients, the EF was available for counseling about the choice. Outcome measures were the percentage of engaged patients among the total population, the percentage of active interventions versus PPA and their distribution among the available options, as well as the factors related to patients’ choices (anthropometry, clinical, exercise capacity, and others). **Results**: Of the 297 eligible patients, 221 met the inclusion criteria, 176 (59%) of whom chose to participate (males, *n* = 113; aged 68 ± 10 years). The patients’ choices were as follows: S-SU-LI, (n = 80), S-HB-LI (n = 66), PPA (n = 25) and U-PA-I (n = 5). Patients in the LI group were significantly older (*p* < 0.001) and had a lower exercise capacity, as measured by the 6 min walking distance (*p* < 0.001). No significant differences in sex, distance from the dialysis center, dialysis duration, or recruiting site were recorded. The main reasons for nonparticipation were not being interested (67%) or already active (22%). **Conclusions**: In this exploratory research, patients exhibited positive attitudes toward exercise training and abandoning a sedentary lifestyle when counseled by an EF and when offered the possibility to choose. As the patient profile becomes more comorbid and frail, supervised and/or lower-intensity programs are preferred.

## 1. Introduction

Chronic kidney disease (CKD) represents a significant global health burden, affecting approximately 10% of the population and increasing in prevalence in low-income countries [1,2].

The dialysis population continues to grow, driven by broader treatment access for elderly patients and a persistent shortage of available transplants [3,4]. Furthermore, the end stage of disease is considered a disease of premature aging, with patients considered physiologically older even by age 50 [5,6,7,8]. Hemodialysis treatment is often associated with sedentary behavior, sarcopenia, and psychological distress, which collectively diminish quality of life [9]. This progressive decline in physical fitness and muscle strength further increases the risk of falls and hospitalizations [10,11,12,13,14]. These factors ultimately create a high-risk subpopulation characterized by severe functional impairment, significant socioeconomic costs, and increased mortality [6,8,15].

This physical decline must be counteracted to reduce deconditioning, cardiovascular risk and mortality [14,16,17,18]. Accordingly, guidelines recommend that nephrologists counsel patients with end-stage kidney disease (ESKD) and regularly encouraged to increase their level of physical activity [2,19]. For over 40 years, exercise has been recognized as an important adjunct in kidney disease treatment, with numerous trials demonstrating the effectiveness of various interventions [20,21,22,23,24,25,26]. Research has investigated both supervised exercise programs, primarily during hemodialysis (HD) sessions [27,28,29,30,31,32,33,34,35,36,37,38,39,40,41,42,43,44], and home-based alternatives [45,46,47,48,49]. While intradialytic cycling is widely available despite patient concerns [50], home-based programs have gained interest in recent years as a means to overcome barriers [46,51], and have demonstrated noninferior effectiveness compared with supervised programs [49]. Ultimately, both approaches offer comparable benefits, each with distinct advantages and disadvantages [28,49].

However, barriers to exercise have emerged as widely recognized challenges [14], among both patients and healthcare professionals [50]. These include physical and psychological factors—such as physician approval, social support, patient motivation, and fear—along with local issues such as implementation difficulties in nephrology units and skepticism regarding exercise benefits [14,50,52,53,54]. Qualitative research emphasizes that individualized care and improved provider interaction are crucial [55,56] as well as education, incentives and tailored interventions in the dialysis unit could help overcome some individual barriers [57]. A systematic review identified disease distress, safety concerns, lack of time, and management policies as key causes of low exercise levels [58]. Consequently, actions promoting behavior change and exercise counseling are essential [50], particularly for older adults and transplant candidates who remain poorly engaged in structured exercise [8,59].

Randomized trials have demonstrated that intradialytic cycling combined with kinesiologist counseling improves physical performance and prepares patients for home exercise [43], whereas nurse-led case management significantly enhances physical function and self-perceived health at home [60]. According to the Italian Society of Nephrology, while nephrologists are central to addressing sedentariness, a multidisciplinary team—including nephrologists, nurses, exercise professionals, and dietitians—is necessary for providing comprehensive rehabilitation [61]. Despite these benefits, unfortunately participation remains low, with only 6.9% of patients meeting the recommended physical activity levels [12]. This underscores the need for cost-effective interventions and trials focused on practical implementation [14,50]. Ultimately, transitioning to effective, reproducible programs requires real-life if this necessitates moving beyond the rigid constraints of traditional randomized studies.

This preliminary analysis of a pragmatic trial [62] aims to explore ESKD patients’ preferences and choices about a physical activity program following the incorporation of an exercise professional into six dialysis centers across Europe. The study also aims to assess whether the choice of different exercise options may be mediated by patients’ characteristics and capacities.

## 2. Materials and Methods

### 2.1. Study Design and Settings

This experimental multicentric pragmatic nonrandomized clinical trial was conducted between February 2020 and December 2024. The study was approved by the Area-Vasta Emilia-Romagna Centro Ethics Committee with approval number 48/2019 (date 25 September 2019), and all participants provided written informed consent. The local ethics committees of the other participating centers then approved the study. The complete protocol is publicly available [62], and the trial was registered at Clinicaltrials.gov with record number NCT04282616(registion date: 20 February 2020). The study is reported according to the TREND guidelines [63].

The study, which was designed as a single-center trial but became a multicenter clinical trial, was conducted at six dialysis centers in three different countries (Italy, Spain, France), including University Hospital of Ferrara, Pederzoli Hospital Dialysis Unit in Peschiera del Garda; Hospital Universitario Reina Sofia, Cordoba; and Centre Ospitalier Carcassone Dialysis Unit.

### 2.2. Inclusion Criteria

Patients with CKD at KDIGO stage V (estimated glomerular filtration rate < 15 mL/min/1.73 m^2^) who had undergone hemodialysis for at least 3 months in stable condition were invited to participate if they were greater than 18 years old, able to walk for at least 6 m and had a Mini Mental Status Examination score ≥ 18/30 [64] to provide informed consent.

The exclusion criteria were uncorrected anemia (hemoglobin concentration < 9 g/dL); acute infectious or inflammatory disease (C-reactive protein > 10 mg/L); and severe cardio-respiratory, musculoskeletal or neurological conditions inhibiting exercise training.

Nephrologists promoted the intervention enabling patients to participate on the basis of their physical condition and the low or moderate intensity of the proposed exercise.

### 2.3. Intervention: First Contact with ESKD Patients

As described in the published protocol [62], an exercise facilitator (EF) with specific experience in diseased populations was set in each dialysis center. During the initial phase of their stay at the dialysis center, after interacting with healthcare staff and patient representatives, EFs initiated contact with the dialysis center population, performing unstructured interviews with ESKD patients.

During these meetings, the EFs explored patients’ attitudes (including feelings, fears, barriers, etc.) toward improving their lifestyle on the basis of physical activity and exercise, their availability of time, and, finally, their interest in possibly participating in programs made available by the dialysis center.

The EF then proceeded to present the designed programs, describing their characteristics and possible benefits, answering patients’ questions or concerns, and providing informative material about the study. Finally, the EF collected either positive or negative comments on possible active participation in the project and gathered registrations to start planning the sessions to be agreed upon with the patients themselves.

After approximately one week, the EF returned, asking which kind of option they would like to choose from the four options to participate in, as described below. If patients asked the EFs for advice to select among the options, each EF was trained to report the advantages and potential disadvantages of each proposal, but the final decision was left to each patient.

### 2.4. Intervention: Training Options

On the basis of previous experiences and the literature, the researchers identified four forms of possible active participation. Aspects common to the treatments were the lack of need for special equipment, low management costs, and implementation without aggravating or modifying the routine and workload of the dialysis center staff. The aim was to enable easy implementation and widespread dissemination in different locations; therefore, the proposed interventions were either in-dialysis or home-based.

The implementation of an intradialysis program based on cycling during dialysis could be activated in response to a significant number of requests (>equal to 20% active), considering that this effective method of physical exercise, provided in various international locations, fell outside the above criteria.

The proposed interventions, which could be carried out at the person’s discretion, were as follows:

- Unstructured physical activity (U-PA-I): In accordance with the guidelines for physical activity for people on dialysis, advice was given on recommended amounts of exercise, although no structured programs were provided for implementation [65]. This intervention required no specific material;
- Structured home-based low-intensity exercise (S-HB-LI): According to each patient’s baseline physical activity level, a semipersonalized walking program was provided composed of a 1 min walking:1 min resting ratio to be repeated 6 or 8 times according to the patient’s baseline functional capacity. The training speed, which was maintained through a metronome, progressively increased throughout the program [46,66]. The equipment for this option was represented by a metronome, in the form of an instrument or a smartphone application that was downloadable at no cost;
- Structured supervised low-intensity exercise (S-SU-LI): Patients met at a specified location (room or corridor) into the dialysis unit for the exercise program in groups of a maximum of four subjects. The thirty-minute training sessions were scheduled on a 2- or 3-time/week pattern, and they were performed immediately before or after the dialysis treatment or according to patients’ preferences and considering the postdialysis fatigue that affects this population [67]. Each exercise session was composed of a low-intensity interval walking component and a strengthening component for the lower and upper limb muscles. The training intensity progressed throughout the program [62]. The equipment, in addition to the metronome reported above, required four ankle weights of different loads;
- Physical performance assessment only (PPA): Patients who chose this option did not begin any exercise program, but they performed the outcome measures session only. A switch to another treatment, periodically proposed by the EF, was always possible.

All the training programs lasted for three months. Details about each exercise program are reported in the study protocol [62].

### 2.5. Intervention: Outcome Measures

Outcome data were collected at baseline (T0), after the end of the three-month training period (T3), and when possible at the 6-month follow-up (T6) by blinded assessors. Blinding may have been compromised by the patient, who, during some of the outcome measures, may have revealed his/her choice.

The following outcome measures were collected for each patient.

The primary outcome of the project was the 6 min walking test. The subjects were asked to walk as far as possible in 6 min via their usual walking aid, if it was used. The test was performed in a 22 m corridor. Patients were allowed to slow down and rest if necessary. The total distance covered, the distance covered without pain and the perceived exertion at the end of the test (Borg scale 1–10) were recorded [68].

In addition, the following measures were taken:

- Gait speed through the 10 m walk test. The subjects were asked to walk as fast as possible over a distance of 10 m, without putting themselves at risk, and to use any walking aids if necessary. The time taken to cover the central 6 m was recorded. The test was repeated twice, and the best result was reported [69];
- Lower limb strength was measured by the 5-time sit-to-stand test (5STS). Patients were placed in a standard chair and asked to stand up and sit down from the chair five times as quickly as possible without using their arms for support. The data collected were the time between the first stand-up and the moment when the subject’s glutes touched the chair for the fifth time [70];
- Health-related QoL was measured by the Italian version of the Short Form Health Survey (SF-36). The questionnaire, which was administered to patients during dialysis sessions, consists of 36 questions that explore how much a person’s health status impacts their perceived quality of life. The results are expressed in eight domains, each with a score from 0 to 100, concerning aspects related to physical and mental health [71];
- Fear of falling was assessed through the Short Falls Efficacy Scale (S-FES-I). This tool consists of seven items that measure the subject’s perceived fear of falling while performing different activities. The total score ranges from 7 to 28, with higher values corresponding to greater fear of falling [72];
- Depression was evaluated by the Beck Depression Inventory-II (BDI-II). This questionnaire consists of 21 items that measure the subject’s level of depression through a series of questions related to the previous two weeks. The score ranges from 0 to 63, with the degree of depression increasing as the score increases [73];

All the questionnaires and scales were administered in the local language through their validated versions. For the purpose of this study, many other data were collected to pursue the aim of this exploration study, including distance from the patient’s home to the dialysis center, ability to drive autonomously toward the center or necessity to be accompanied and dialysis vintage.

### 2.6. Sample Size

As a pragmatic, exploratory study, we calculated the sample size on the basis of the number of dialyzed patients in the different centers (*n* = 200). According to a power calculation based on a theoretical responder rate of 50% and considering a margin of error equal to 5% and a confidence level of 95%, a total of 132 patients will be required to make the first qualitative phase of the study representative of the population.

### 2.7. Statistical Analyses

The data distribution was verified through the Kolmogorov—Smirnov test. Descriptive statistics have been employed to describe the patient population, reporting data as the mean or median (95% confidence interval) or number and percentage according to the data nature and distribution.

Comparisons between the patients’ choices of the different possibilities were performed via independent samples analysis of variance or Kruskal—Wallis tests for continuous variables with Bonferroni’s corrected post hoc analyses. Chi-square tests were employed for categorical variables. Effect size values for multiple comparisons, together with 95% confidence interval when available, were reported through η^2^ or Cremer’s V values accordingly. Multinominal logistic regression models were employed to assess the presence of confounding factors. A *p* value < 0.05 was considered statistically significant. Data analyses were performed with MedCalc^®^ Statistical Software version 23.4.8 (MedCalc Software Ltd., Ostend, Belgium) and SPSS version 30.1 (IBM, New York, NY, USA).

## 3. Results

A total of 297 patients on maintenance HD were screened for eligibility. The mean age was 72.5 ± 13.7 years, and 33% (*n* = 97) were women. A sample of 76 patients did not meet the inclusion criteria, mainly for inability to walk (*n* = 57), cognitive decline (*n* = 12) or other clinical conditions preventing participation (*n* = 7). A final sample of 221 patients was approached by the EFs.

### 3.1. Contact with the Eligible Patients

All eligible patients had a meeting with the EFs at their dialysis unit. The different types of responses in favor of or against participation collected are shown in Table 1. Among the 221 patients eligible for the study, 45 decided not to take part in the study. The main reasons for refusal were a general lack of interest in exercise and physical activity (*n* = 30), being already physically active (*n* = 10), undergoing physiotherapy sessions (*n* = 2) or, finally, a lack of time to exercise (*n* = 3) (Table 2).

Among the patients who agreed to participate, only four expressed interest in participating in an intradialytic cycling program during the treatment. This option has therefore not been activated due to insufficient interest.

A final sample of 176 patients agreed to participate in the study. The population that decided not to participate did not present any significant difference within the population that agreed to participate in the study (Table 3).

**Figure 1:**
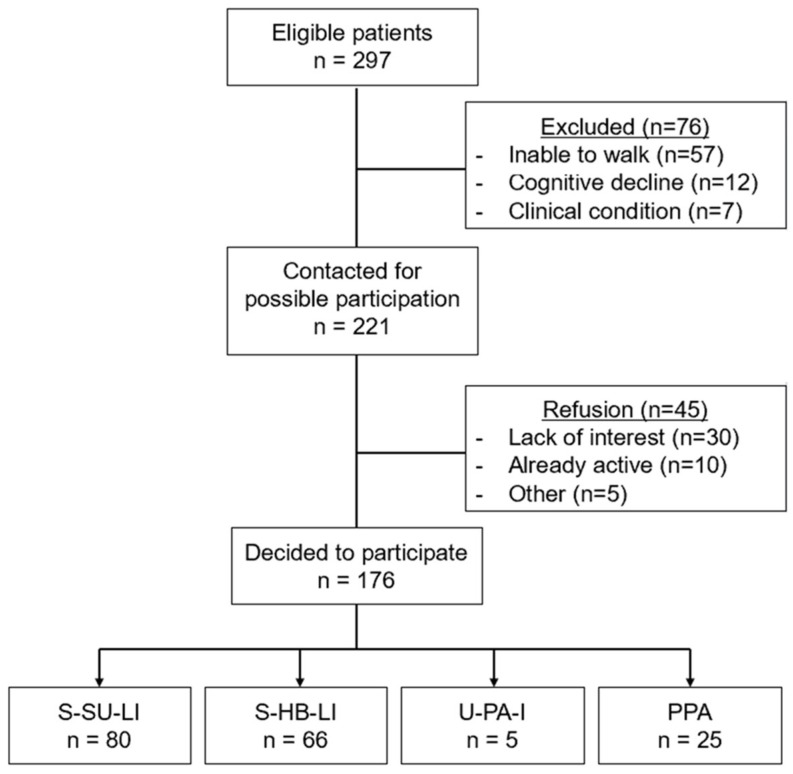
Flow chart of the study.

The enrollment process of the entire study is reported in Figure 1.

### 3.2. Factors Associated with Patients’ Choice of Different Active Programs

Among the 176 participants, 80 (45%) opted for the S-SU-LI supervised intervention, 66 (38%) chose the S-HB-LI program, 25 (14%) chose the performance assessment only, and 5 (3%) chose the U-PA-I.

### 3.3. Age and Sex

Patients in the S-SU-LI intervention group were significantly older than those in all the other groups, with a mean difference ranging from 8 to 16 years. No other between-group significant differences were noted, and no differences were noted between the sexes.

### 3.4. Dialysis History and Comorbidities

Patients who chose the U-PA-I option had a significantly greater duration of dialysis, followed by those in the S-SU-LI group; the S-HB-LI group had the shortest duration since the beginning of dialytic treatment. When considering comorbidities, patients opting for the S-SU-LI and S-HB-LI programs showed significantly greater Charlson comorbidity index scores than did those in the other two groups.

### 3.5. Transportation

Interestingly, patients who chose the S-SU-LI intervention, who were also older than those in the other groups, were significantly more likely to use caregiver or transport services to the dialysis centers, whereas the majority of patients in the PPA and U-PA-I groups drove themselves to the hospital.

Finally, no differences in terms of European countries were associated with patients’ choices.

### 3.6. Level of Physical Functioning at Entry

At entry, people who chose S-SU-LI exercise presented a significantly lower exercise capacity in terms of distance walked with respect to all the other groups, with a 6MWD lower than 61 m with respect to S-HB-LI, 34 m with respect to U-PA-I, and 117 m with respect to the PPA group.

Patients in the S-SU-LI and S-HB-LI groups also presented a slower gait speed and a lower leg strength than did those in the PPA group. Finally, a greater fear of falling was present in the S-SU-LI group. The data are reported in Table 4.

### 3.7. Depressive Symptoms and Quality of Life

Compared with both the S-HB-LI and PPA groups, the S-SU-LI group presented greater depressive symptoms.

In addition, when considering quality of life, significant differences were observed for the S-SU-LI group in terms of physical functioning scores (which were lower than those of the S-HB-LI and PPA groups) and role-emotional scores (which were lower than those of the S-HB-LI). Overall, the choice of S-HB-LI and PPA groups was associated with greater values of perceived health-related quality of life than the other groups were, although the difference was not statistically significant. The data are presented in Table 5.

A multinominal logistic regression model was built using the four different training options as independent variables and all the other factors as dependent variables or covariates. Unfortunately, due to the very low sample size in the U-PA-I group and the lack of some characteristics in some groups, the created model was invalid.

## 4. Discussion

The study highlighted a feasible implementation of a simple intervention in various European locations on the basis of the presence of an EF approaching the dialysis population and offering simple activities or training programs with different features. This approach was associated with a very satisfactory overall participation rate, confirming the potential interest of the ESKD patients in improving their lifestyle through physical activity. As is often the case in real-world studies, this original pragmatic feasibility trial offers useful insights and food for stakeholders, which we describe in the following paragraphs.

In activities for special populations, personalization (program design on the basis of skills, abilities and preferences), task presentation, and the environment are critical factors [74]. On this basis, the novelty of this study is the combination of an onsite exercise physiologist (EF) in a dialysis center, which helps with the choice of exercise program with different characteristics in terms of time, location and methods of participation.

Few studies have employed trained healthcare professionals [EFs or physiotherapists] to both counsel and supervise exercise in the ESKD population [43,58,59], despite positive results in other chronic disorders in primary care [75,76,77,78]. For nondialysis patients, the RENEXC study [79] used physiotherapists for individualized training plans, whereas digital facilitators in trials such as BEAM [80] and ENTICE-CKD [81] explored “telehealth coaching”. However, these solutions lack a daily EF presence in the dialysis unit, creating a small but present barrier to participation.

Indeed, physical activity levels in CKD patients are remarkably low, with only 6–34% reported activity via validated questionnaire [82], dropping to 6% in hemodialysis patients [83]. Even in clinical trials, which typically enroll only half of the target population [84], adherence after one month ranges from 40 (home-based) to 70% (intradialytic) [32,56,84]. Consequently, high-quality clinical trials often find that only 20–35% of patients effectively adopt exercise programs. In this context, a 79% participation rate observed in the study following interaction with the EF is significant, especially considering that an additional 5% declared themselves as already active. Several factors likely favor this response. First, minimal exclusion criteria allowed participation for all patients except those with absolute contraindications. This was made possible by the gentle, progressive training stimuli, even for home-based components [45], monitored by the EF in collaboration with nephrologists. Second, allowing patients to select their preferred training option increased participation. While CKD-specific preference trials are lacking, evidence from chronic disease shows that assigning exercise instead of offering choice nearly doubles dropout rates [50% vs. 27%] [85]. Furthermore, an “internal locus of control” is a primary predictor of long-term adherence [70% vs. 50%] [26]. Finally, self-determination theory (SDT) confirms that “autonomous motivation” (choice-driven) is the only significant predictor of long-term exercise adherence in CKD patients, whereas “controlled motivation” (physician-assigned) leads to high long-term dropout [86,87].

A second highlight of this study was the patient selection of proposed interventions. Factors such as age, comorbidities, perceived functioning, and logistics (e.g., transport and distance) influenced these choices, particularly with respect to structured exercise. Only a minority (n = 5), typically younger and less comorbid patients, opted for walking advice, as suggested by guidelines. We can only speculate on the reasons underlying these choices, but the amount of time to be spent daily on physical activity (from 10 to 30 min) likely represented the main barrier perceived by the majority of the patients, as reported in the literature [88]. The low-intensity structured home-based exercise (S-HB-LI), which has proven effective in other frail populations [45,49], was chosen by the second-eldest group, which also had the longest travel times. Interestingly, the eldest and most comorbid subgroup opted for supervised intervention. Since the aerobic intensity mirrored the S-HB-LI group, other factors were likely associated with this choice. First, many in this group used transport services that arrived 30–60 min early, allowing them to utilize waiting time for exercise. Second, their higher levels of disability, comorbidity and older age may have created perceived barriers and fears that necessitated a training supervisor to overcome.

Choosing an appealing, well-presented program is crucial. Despite evidence supporting exercise in daily routines [14] and large-scale studies demonstrating feasibility [31,45,89,90], real-world implementation remains low, with existing models focused primarily on home-based or intradialytic cycling [28,91]. Therefore, this pragmatic study reported both the feelings and thoughts of participants and showed that while fitter patients preferred recommendations for independent exercise, as the patient profile becomes more comorbid and frail, it is necessary to adapt programs to lower intensities or provide supervised execution. Finally, which is not surprising but differs from previous observations in the literature, with the freedom to choose, patients demonstrate a positive attitude toward physical exercise, at least at an initial stage.

This study has several limitations. First, a cycling training option during dialysis treatment was not offered, despite the evidence in the literature [84,91,92]. However, we aimed to test training solutions requiring minimal space and equipment that are implementable in most dialysis centers worldwide and not overtaxing personnel or the routine of a dialysis center. Interestingly, in this direction, only four respondents [9] said they would participate in a dialysis cycling program. The others expressed interest in combined low-to-moderate intensity aerobic and resistance programs carried out during dialysis if they were particularly frail or if they were tied to transport. A second limitation was that the study was designed as a single center but was then spread to four additional centers to improve its statistical power. Additionally, despite having involved a significant number of patients, their preferences for exercise may reflect only a partial side of Europe (southern). In addition, the small sample of patients who chose the U-PA-I group may represent a selection bias that has influenced the analyses, making it also impossible to perform a multinominal logistic regression. Finally, the effect of dialysis modality on physical activity was not assessed although a recent EuDial consensus statement suggests patients on HD may be more active than those on high-flux HD [93].

## 5. Conclusions

In conclusion, this pragmatic study in a real-world European setting explored ESKD patients’ feelings towards exercise training, finding a positive attitude when they were given the freedom to choose. Since no differences were found among the participating European countries and considering that the proposed model does not require particular equipment but only qualified personnel, this simple exercise program could potentially be implemented in each dialysis unit worldwide.

The continuation of the study will evaluate actual patient adherence to the selected program and determine whether one intervention is more effective than the other.

## Figures and Tables

**Table 1:** Examples of positive and negative comments about the attitudes and feelings of patients toward exercise.

Answer
I knew I needed to exercise, but I did not know who to turn to. Without EF at the dialysis center, I would not have been able to train at home or at the gym.
I am already physically active, but I believe that the opportunity to perform exercises that I would not do on my own, under the supervision of the EF, is an opportunity to be seized.
Due to my disease, I thought I would not be able to follow any exercise program. The opportunity to engage with an expert on a daily basis helped me find an exercise program that was compatible with my condition.
I think I have good autonomy, but I am glad to be able to undergo free assessment sessions at the dialysis center.
I do not truly like exercising. However, talking to EF helped me identify some exercises that can benefit me and that I enjoy doing.
I would have liked to do supervised exercise, but due to transportation issues, this is impossible for me. I appreciated that EF was able to give mean exercise program to do at home, which I can follow despite my limitations.
I was interested in being independent in my household activities, and I did not want to start any very demanding programs. The prospect of gaining independence with little effort seemed appealing to me.
I have some friends who go to the gym, but the prospect of training with more active people has always discouraged me. The opportunity to exercise under the supervision of an expert, together with other people in my condition, seemed like an ideal situation.

**Table 2:** Main reasons for nonparticipation given by the patients.

N	Females, n (%)	Age, Mean (SD)	Reason
1	0 (0)	74 (n.a.)	I am incapable of following a program at home, and I am unable to participate in a supervised program.
1	1 (100)	78 (n.a.)	I am seeing a physiotherapist privately with benefits, and I do not feel the need to participate in the program.
10	1 (10)	66.3 (16.1)	I consider myself sufficiently active, and I do not believe I need to increase my level of physical activity.
3	1 (33)	63.7 (24.4)	I do not have enough free time to exercise.
30	9 (30)	77.0 (11.8)	I do not think I need to exercise/I do not believe my health would improve by participating in the program.

Legend: n.a., not applicable

**Table 3:** Baseline comparisons of patients who agreed to participate in the study versus those who did not.

	Participants (*n* = 176)	Non Participants (*n* = 45)	*p* Value
Age	70.0 ± 12.8	72.5 ± 14.9	0.12
Male sex, *n* (%)	124 (71)	33 (73)	0.82
Dialysis vintage, years	7 ± 3	7 ± 4	0.74
Distance from dialysis center, km	19.5 ± 16.5	21.8 ± 15.6	0.43
Cardiovascular disease	132 (75)	32 (71)	0.67
Stroke	21 (12)	4 (9)	0.23
Pulmonary disease	25 (14)	5 (12)	0.45
Rheumatic disorder	32 (18)	6 (16)	0.82
Cancer	58 (33)	16 (35)	0.77
Charlson Index *	5.6 ± 4.1	5.8 ± 3.9	0.88

* The Charlson comorbidity index was not age-adjusted.

**Table 4:** Baseline comparisons of participating patients according to their choice of exercise program.

	S-SU-LI (n = 80)	S-HB-LI (n = 66)	U-PA-I (n = 5)	PPA (n = 25)	*p* Value	Effect Size (95% CI)
Age	74.5 ± 10.6	66.7 ± 13.5 *	58.6 ± 8.0 *	63.7 ± 12.0 *	<0.001	0.124 (0.015–0.225)
Male sex, n (%)	54 (68)	50 (76)	3 (60)	20 (80)	0.49	0.105
Dialysis vintage, years	7.6 ± 3.1	5.9 ± 2.4 *	8.3 ± 8.0†	6.6 ± 6.2	0.032	0.072 (−0.022–0.175)
Charlson Index	6.4 ± 2.1	5.8 ± 1.9 †	4.3 ± 1.5 * †	4.0 ± 1.5 * †	0.006	0.099 (−0.021–0.215)
Distance from dialysis center, km	17.4 ± 13.9	20.3 ± 14.7	14.8 ± 11.8	16.5 ± 13.6	0.44	−0.022 (−0.033–0.017)
Mean of transportation		*	* † ‡	*	0.002	0.160
Autonomous	24 (27)	30 (50)	5 (100)	12 (48)		
Caregiver/Service	58 (66)	18 (30)	0 (0)	10 (40)		
Public	6 (7)	12 (20)	0 (0)	3 (12)		
Italy/Europe	53/27	44/22	4/1	17/8	0.86	
Outcome measures						
6MWD, m	251 ± 107	316 ± 122 *	285 ± 154	368 ± 126 *	<0.001	0.105 (0.024–0.187)
T10m, s	7.52 ± 4.32	6.98 ± 7.54	10.54 ± 8.29	4.17 ± 1.80 * †	0.10	0.048 (0.000–0.112)
5STS, s	16.71 ± 10.72	15.11 ± 6.64	14.73 ± 5.23	12.18 ± 3.11 * †	0.28	0.041 (−0.021–0.087)
S-FES-I	11.7 ± 5.4 †	9.3 ± 3.4	10.8 ± 3.8	11.3 ± 10.3 †	0.26	0.084 (0.000–0.182)
DASI	24.4 ± 15.0	33.9 ± 15.5 *	27.7 ± 14.0	33.6 ± 18.0 *	0.14	0.118 (0.001–0.234)

* *p* < 0.05, then S-SU-LI; † *p* < 0.05, then S-HB-LI; ‡ *p* < 0.05, then PPA; *p* < 0.05, then U-PA-I. Effect size: η2 for continuous variables; Cremèr’s V for categorical variables. Legend: 6MWD, 6 min walking distance; T10m, timed-10 m test; 5STS, 5-time sit-to-stand test; S-FES-I; Short Falls Efficacy Scale Italian; DASI, Duke Activity Status Index; CI, confidence interval.

**Table 5:** Comparisons of quality of life parameters collected at baseline in participating patients according to their choice of exercise program.

	S-SU-LI (n = 80)	S-HB-LI (n = 66)	U-PA-I (n = 5)	PPA (n = 25)	*p* Value	η2 (95% CI)
BDI-II	10.7 ± 2.0	9.2 ± 2.1 *	9.5 ± 2.2	9.0 ± 1.8 *	0.052	0.823 (0.000–0.187)
SF-36 questionnaire domains						
Physical functioning	59 ± 28	73 ± 19 *	50 ± 41	77 ± 18 *	0.011	0.106 (0.005–0.210)
Role-Physical	57 ± 36	61 ± 36	38 ± 43 †	48 ± 47	0.52	0.020 (0.000–0.075)
Role-Emotional	67 ± 36	85 ± 33 *	50 ± 58 †	67 ± 41	0.053	0.076 (0.000–0.170)
Vitality	54 ± 19	57 ± 16	35 ± 33	60 ± 15	0.08	0.068 (0.000–0.159)
Mental Health	68 ± 20	71 ± 14	60 ± 24	72 ± 23	0.64	0.016 (0.000–0.066)
Social functioning	64 ± 28	72 ± 24	47 ± 48	70 ± 28	0.25	0.041 (0.000–0.117)
Bodily pain	69 ± 31	72 ± 20	62 ± 34	73 ± 29	0.82	0.009 (0.000–0.043)
General health	41 ± 20	45 ± 15	26 ± 9	43 ± 28	0.24	0.042 (0.000–0.119)

* *p* < 0.05, then S-SU-LI; † *p* < 0.05, then S-HB-LI; *p* < 0.05, then U-PA-I. Legend: BDI-II, Beck’s Depression Inventory-II; SF-36, short-form 36 questionnaire; CI, confidence interval.

## Data Availability

The data supporting the findings of this study are available from the corresponding author upon reasonable request. Due to the ongoing nature of further investigation, some datasets are currently unavailable for public access.

## Abbreviations

The following abbreviations are used in this manuscript:

EF	Exercise facilitator
ESKD	End-stage kidney disease
U-PA-I	Advised physical activity increase
S-HB-LI	Structured home-based walking exercise
S-SU-LI	Supervised walking and resistance low-intensity training
PPA	Performance assessment only
CKD	Chronic kidney disease
HD	Hemodialysis
5STS	5-time sit-to-stand test
SF-36	Short Form Health Survey
S-FES-I	Short Falls Efficacy Scale
BDI-II	Beck Depression Inventory-II
SD	Standard deviation
n.a.	Not available
6MWD	Six-minute walking distance
T10m	Ten-meter walking test
DASI	Duke’s activity status index
SDT	Self-determination theory
